# Anwendungspraxis der apnoischen Oxygenierung in der Anästhesiologie: eine deutschlandweite Umfrage

**DOI:** 10.1007/s00101-025-01529-2

**Published:** 2025-04-28

**Authors:** Davut Deniz Uzun, Felix Hezel, Stefan Mohr, Markus A. Weigand, Felix C. F. Schmitt

**Affiliations:** Medizinische Fakultät Heidelberg, Klinik für Anästhesiologie, Universität Heidelberg, Im Neuenheimer Feld 420, 69120 Heidelberg, Deutschland

**Keywords:** Anästhesie, Narkose, Präoxygenierung, Sauerstoff, Atemwegssicherung, Patientensicherheit, Anesthesia, Narcosis, Preoxygenation, Oxygen, Airway management, Patient safety

## Abstract

**Hintergrund und Fragestellung:**

Die apnoische Oxygenierung (ApOx) stellt ein Verfahren dar, welches im Rahmen der Narkoseeinleitung zur Verlängerung der sicheren Apnoezeit sowie zur Vermeidung von Desaturation zum Einsatz kommt. Trotz Erwähnung in nationalen sowie internationalen Leitlinien ist die praktische Anwendung der ApOx in Deutschland bisher nicht wissenschaftlich erfasst.

**Ziel der Arbeit:**

Die vorliegende Studie verfolgt das Ziel, die praktische Anwendung der ApOx in deutschen Kliniken aller Versorgungsstufen zu evaluieren.

**Material und Methoden:**

Zwischen 01.07.2024 und 03.09.2024 erfolgte eine multizentrische, explorative, anonyme Befragung der Anästhesisten in verschiedenen deutschen Kliniken. Die Befragung erfolgte über die Online-Plattform Lime Survey (Hamburg, Deutschland). Die Auswertung erfolgt auf Basis einer deskriptiven Statistik.

**Ergebnisse:**

Die Umfrage wurde von insgesamt *n* = 310 Anästhesisten bearbeitet; nach Ausschluss der unvollständigen Datensätze konnten *n* = 306 Fragebogen analysiert werden. Die Mehrheit der Teilnehmer war männlich (63 %), mit einem Durchschnittsalter von 41 Jahren (± 10,4). Die Verteilung der Karrierestufen zeigte das folgende Bild: Oberarzt (34 %), Arzt in Weiterbildung (34 %), Facharzt (25 %), Chefarzt (8 %). In 84 % der Fälle konnte festgestellt werden, dass weder innerklinisch noch prähospital eine Standardarbeitsanweisung zur ApOx existierte. In der vorliegenden Studie gaben 18 % der Anästhesisten an, die ApOx regelmäßig durchzuführen. Die Anwendung erfolgt in den folgenden Situationen: 54 % „respiratorische Insuffizienz“, 42 % „erwartet schwieriger Atemweg“ und 34 % „Notfallnarkose“. Die praktische Durchführung erfolgt zu 51 % über eine High-Flow-Nasenkanüle oder zu 33 % über eine Standardnasenbrille.

**Schlussfolgerung:**

Trotz wissenschaftlicher Evidenz, die eine signifikant geringere Hypoxämierate sowie eine erhöhte Wahrscheinlichkeit für einen „First-Pass-Intubation-Success“ bei der Anwendung der ApOx zeigt, findet diese in Deutschland aktuell keine routinemäßige Anwendung. Zum gegenwärtigen Zeitpunkt konnte keine Standardisierung hinsichtlich der Indikation und der Anwendungstechnik der ApOx festgestellt werden.

**Graphic abstract:**

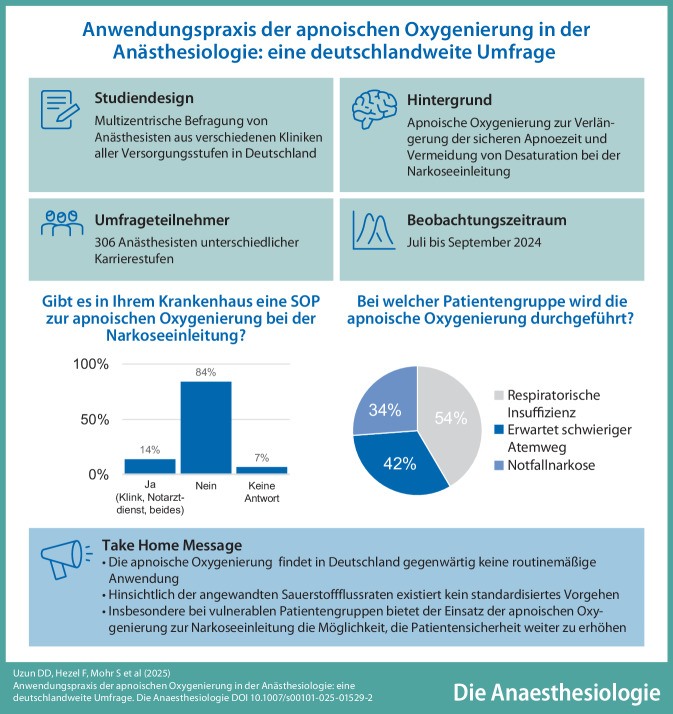

**Zusatzmaterial online:**

Zusatzmaterial zum Beitrag „Anwendungspraxis der apnoischen Oxygenierung in der Anästhesiologie: eine deutschlandweite Umfrage“ von Uzun DD, Hezel F, Mohr S et al. (2025) in: *Die Anaesthesiologie*. 10.1007/s00101-025-01529-2. Beitrag und Zusatzmaterial stehen Ihnen auf www.springermedizin.de zur Verfügung. Bitte geben Sie dort den Beitragstitel in die Suche ein.

## Abstract (en)

**Background and research question:**

While patient safety during general anesthesia has improved in recent years, incidents still occur during anesthesia, particularly in the area of extended airway management. During the induction of general anesthesia, spontaneous breathing is routinely suspended thereby increasing the risk of hypoxemia during the apneic phase. To prevent desaturation and clinically significant hypoxemia, preoxygenation is performed before anesthesia induction. While preoxygenation can extend the safe apnea time, certain high-risk patient groups, such as obese patients, pregnant women, children and those with difficult airways, may require additional stabilization strategies. Apneic oxygenation (ApOx), a technique first described in the early twentieth century, delivers 100% oxygen at a flow rate of 15 l per min or more during the apneic phase to prolong oxygenation. While current airway management guidelines recommend ApOx during the laryngoscopy phase for patients at risk, its widespread utilization in clinical practice remains limited.

**Objective:**

This study presents the findings of a multicenter, web-based survey investigating the use of ApOx in anesthesiology departments across Germany, highlighting the challenges in implementation and identifying areas for further improvement.

**Materials and methods:**

Between 1 July 2024 and 3 September 2024 a multicenter, exploratory, anonymous survey of anesthetists in various German hospitals was conducted. The survey was conducted via the online platform Lime Survey (Hamburg/Germany). Participants were asked about their personal and institutional background and the use of ApOx in their institution The study is based on descriptive statistics.

**Results:**

The survey was completed by a total of *n* = 310 anesthetists and after excluding incomplete data sets, *n* = 306 questionnaires could be analyzed. The majority of participants were male (63%), with an average age of 41 years (± 10.4). In terms of professional roles, there was an even split between senior physicians (34%) and residents (34%), followed by consultants (25%) or heads of departments (8%). The work settings included university hospitals (37%), maximum care hospitals (27%), intermediate care hospitals (19%) and basic and standard care hospitals (15%). The majority of the participants (59%) had an additional qualification in emergency medicine. In addition, 48% of the respondents had an additional qualification in intensive care medicine. In 84% of cases it was found that neither in-hospital nor prehospital standard operating procedures on ApOx exist. Of the respondents 18% indicated that they regularly perform ApOx. The procedure is employed in the following scenarios: 54% for respiratory insufficiency, 42% for expected difficult airway and 34% for rapid sequence intubation. In the clinical practice 51% of procedures utilize a high-flow nasal cannula, while 33% employ a standard nasal cannula.

**Conclusion:**

Despite the scientific evidence indicating that the utilization of ApOx is associated with a markedly reduced incidence of hypoxemia and an elevated probability of first-pass intubation success, it is not employed on a routine basis in Germany. Currently, there is an absence of standardization with respect to the indications and administration technique of ApOx. The underlying factors contributing to this phenomenon are believed to be numerous and multifaceted.

## Hintergrund

Im Rahmen der Induktion der Allgemeinanästhesie kommt es zur Apnoe des Patienten und folglich zunächst zur Unterbrechung der Sauerstoffzufuhr [1, 2]. Um während dieser Apnoephase eine Desaturation und konsekutiv die Entwicklung einer Gewebshypoxie zu vermeiden, soll vor Induktion der Allgemeinanästhesie routinemäßig eine suffiziente Präoxygenierung/Denitrogenisierung durchgeführt werden [3–6]. Während der Präoxygenierung mit 100 %igem Sauerstoff wird die funktionellen Residualkapazität (FRC) rasch denitrogenisiert und mit Sauerstoff aufgesättigt [2, 7]. Die FRC spielt eine entscheidende Rolle für die Wirksamkeit der Präoxygenierung, da der in der FRC „gespeicherte“ Sauerstoff als Reservoir dient, um die Oxygenierung während einer Apnoe aufrechtzuerhalten, wenn kein Sauerstoff zugeführt wird. Auf diese Weise wird die Sauerstoffkonzentration im Alveolarraum maximiert und gleichzeitig die sich im Lungen-Gas-Gemisch befindliche Stickstofffraktion minimiert (Denitrogenisierung) [1, 2]. Angestrebt wird so die Verlängerung der sicheren Apnoezeit, der Zeitspanne, in der ein Patient ohne Beatmung verbleiben kann, bevor die periphere Sauerstoffsättigung (S_p_O_2_) abfällt [7, 8]. Bei kritisch kranken Patienten kann die endotracheale Intubation in bis zu 50 % der Fälle mit lebensbedrohlichen Komplikationen assoziiert sein [9, 10]. Die Ursachen der Instabilität sind in diesem Fall jedoch nicht nur auf eine evtl. auftretende Hypoxämie bei der Narkoseeinleitung zurückzuführen. Im Allgemeinen können Hypotension, kardiovaskuläre Instabilität bis hin zum Herzstillstand sowie zerebrale und renale Gewebehypoxie im Rahmen der Narkoseeinleitung auftreten [10, 11].

Bestimmte Patientengruppen sind während der Induktion der Allgemeinanästhesie einem erhöhten Hypoxämierisiko ausgesetzt [3, 6]. Diese Gefährdung lässt sich auf verschiedene physiologische und anatomische Faktoren zurückführen. Zu diesen Risikogruppen gehören u. a. Patienten mit erhöhtem Sauerstoffbedarf, wie Kinder und kritisch Kranke. Ebenso zählen Patienten mit reduzierter FRC, wie z. B. Schwangere, adipöse Patienten oder pulmonal vorerkrankte Patienten, zu dieser Risikogruppe [12]. Eine weitere Risikogruppe sind Patienten mit schwierigen Atemwegsbedingungen. Hier ist das erhöhte Hypoxämierisiko durch die potenziell verlängerten Apnoephase während der prolongierten Atemwegssicherung begründet und nicht aufgrund des erhöhten Sauerstoffbedarfs [12, 13].

Durch Anwendung der apnoischen Oxygenierung (ApOx) lässt sich die sichere Apnoezeit ohne Desaturation verlängern [3, 5, 6]. Wie bereits in den frühen 1900er-Jahren dargelegt, wird bei der ApOx dem apnoischen Patienten während der Narkoseeinleitung zusätzlich 100 %iger Sauerstoff mit einer hohen Flussrate (beispielsweise 15 l/min) verabreicht [4, 14]. Voraussetzung hierfür ist, dass die oberen Atemwege des Patienten kontinuierlich geöffnet sind, sodass eine Insufflation der Atemwege mit Sauerstoff mit hohem Partialdruck möglich ist [4, 12]. Dabei wird das Prinzip verfolgt, dass durch die Diffusion von Sauerstoff aus den Alveolen in die Blutbahn ein „Sauerstoffunterdruck“ im Alveolarraum entsteht; dieser lässt weiteren Sauerstoff aus dem Atemwegstotraum nachströmen. Der Sauerstoff wird dann wiederum per ApOx kontinuierlich nachgeliefert (Einwärtsdiffusion) [13]. Der Sauerstoffpartialdruck wird durch eine vorausgehende Denitrogenisierung der Lunge mittels Präoxygenierung optimiert, da verbliebener Stickstoff und akkumuliertes Kohlendioxid (CO_2_) die alveolären Gasdruckverhältnisse und somit den Sauerstofffluss in die Alveolen und von dort weiter in die Blutbahn beeinträchtigen können [13, 15]. Das Auswaschen von CO_2_ ist während der ApOx jedoch stark eingeschränkt, was im Verlauf zu einem Anstieg des Kohlendioxidpartialdrucks (pCO_2_) und Abfall des pH-Wertes im Blut (Acidämie) führt [12, 13, 16]. Dieser Umstand begrenzt die Dauer, in der die ApOx sinnvoll angewendet werden kann [13]. Dieser Aspekt ist allerdings im Rahmen der Anwendung der ApOx zur Narkoseeinleitung vernachlässigbar. Für die Anwendung der ApOx sind verschiedene Methoden beschrieben, wie z. B. die Verwendung einer Standardnnasenbrille, eines Rachentubus oder einer High-Flow Nasal Cannula (HFNC) [13]. Eingesetzt werden sowohl ungewärmter und trockener Sauerstoff, beispielsweise beim Verfahren „nasal oxygen during efforts at securing a tube“(NODESAT, [13]), als auch transnasal befeuchtete Techniken, wie der transnasal befeuchtete Beatmungsaustausch mit schneller Insufflation („transnasal humidified rapid-insufflation ventilatory exchange“, THRIVE, [17]). Die Wirkung der ApOx kann beispielsweise durch kollabierende obere Atemwege sowie durch unzureichende Präoxygenierung, die zu suboptimalen Gaspartialdrücken im Alveolarraum führt, eingeschränkt sein [18]. Bereits seit einigen Jahren erwähnen die gültigen Leitlinien zum Atemwegsmanagement die Anwendung der ApOx im Rahmen der Narkoseeinleitung insbesondere bei hypoxämiegefährdeten Risikogruppen [3, 4, 15, 19]. Auch in der aktuellen S1-Leitlinie zum Atemwegsmanagement der Deutschen Gesellschaft für Anästhesiologie und Intensivmedizin (DGAI) findet die ApOx bereits Erwähnung. Allerdings wird hier keine Empfehlungen zur Art und Weise der Anwendung von ApOx im Rahmen der Narkoseeinleitung des Erwachsenen beschrieben [5]. Obgleich die ApOx seit den 1900er-Jahren bekannt ist, liegen aktuell keinerlei wissenschaftliche Informationen über deren Anwendung in deutschen Kliniken vor. Ziel dieser Arbeit ist es daher, die Anwendungsindikationen, Häufigkeiten sowie Methoden der ApOx in der klinischen Praxis zu evaluieren.

## Material und Methoden

Diese multizentrische Studie wurde in Übereinstimmung mit den institutionellen Richtlinien und der Deklaration von Helsinki von 1975 in ihrer neuesten Fassung durchgeführt. Eine ethische Genehmigung dieser Studie wurde von der lokalen Ethikkommission (Universität Heidelberg, Medizinische Fakultät Heidelberg, Deutschland) als nicht erforderlich erachtet, da keine identifizierbaren Daten vorhanden sind. Korrespondenz am 07.06.2024 (§ 15 Abs. 1 BOÄ BW). Diese Studie entspricht den Standards der STROBE-Erklärung [20].

### Studiendesign und Teilnehmerauswahl

Im Rahmen der multizentrischen, webbasierten und anonymen Umfrage wurden Anästhesisten in verschiedenen deutschen Krankenhäusern auf allen Versorgungsebenen befragt. Die Teilnehmer konnten über einen anonymen (nichtpersonalisierten) Weblink auf die Umfrage zugreifen. Nach Bestätigung der Datenschutzbestimmungen der Umfrage konnten sie sofort mit der Beantwortung der Fragen zur ApOx beginnen. Die Einschlusskriterien lauteten:

Die Teilnehmer müssen über 18 Jahre alt sein und entweder Arzt in Weiterbildung oder Facharzt für Anästhesiologie sein.

**Abb. 1:**
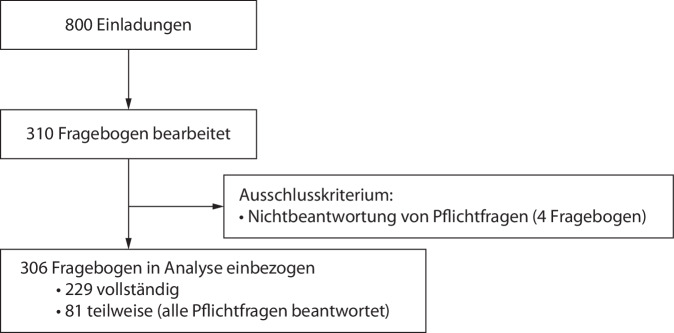
Flow-Chart zum Teilnehmer-Recruitment

**Abb. 2:**
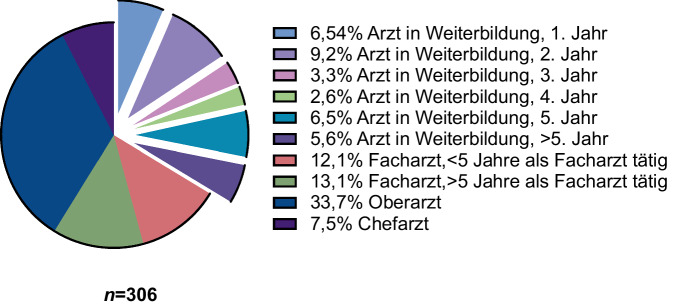
Verteilung der Karrierestufen in der Studienpopulation

Das Ausschlusskriterium lautete: Nichtbeantwortung von Pflichtfragen durch den Teilnehmer. Als Pflichtfragen wurden vor Beginn der Studie die Fragen 3 und 5 festgelegt. Diese standen am Anfang des Fragebogens und erfassten den Ausbildungsstand des Anästhesisten (Frage 3) sowie das Vorhandensein von Zusatzbezeichnungen (Frage 5). Nach Ausschluss unvollständiger Umfragen wurden 306 von insgesamt 310 Teilnehmerin die endgültige Analyse einbezogen, wie in Abb. 1 dargestellt. Abb. 2 zeigte die Verteilung der Karrierestufen in der Studienpopulation.

Im Rahmen der empirischen Untersuchung zur Anwendung der ApOx in der Praxis wurde ein Fragebogen mit 16 Items konzipiert. Mit dem Ziel einer Optimierung der Qualität des Instruments wurden die Items einem modifizierten kognitiven Testverfahren unterzogen. Der ursprüngliche Fragebogen wurde durch semistrukturierte Interviews mit 25 Ärzten unterschiedlichen Ausbildungshintergrunds evaluiert. Des Weiteren wurde der Fragebogen einer Prüfung durch einen Experten für medizinische Ausbildung unterzogen. Im Anschluss an die Integration des Fragebogens in die Online-Plattform (LimeSurvey, Hamburg, Deutschland) erfolgte eine zweite Runde der Überprüfung der Fragen durch ein Gremium aus 25 medizinischen Expertinnen und Experten. Die Datenerhebung erfolgte im Zeitraum vom 01.07.2024 bis zum 03.09.2024. In der Folge wurde der Weblink deaktiviert.

Der Weblink zur Umfrage wurde an alle registrierten Mitglieder des Wissenschaftlichen Arbeitskreises Atemwegsmanagement der DGAI übermittelt. Des Weiteren wurden Universitätskliniken und andere Krankenhäuser sämtlicher Versorgungsstufen dazu eingeladen, sich elektronisch an der Umfrage zu beteiligen. Dazu wurden die Abteilungsleiter der Kliniken kontaktiert und gebeten, den Weblink in ihren jeweiligen Abteilungen zu kommunizieren. Insgesamt wurden etwa 800 Anästhesisten zur Teilnahme an der Umfrage eingeladen. Eine Erinnerungs-E-Mail wurde nicht versandt.

### Statistische Auswertung

Die erhobenen Daten, die der Auswertung zugrunde liegen, wurden mithilfe von deskriptiver Statistik analysiert. Diese erfolgte unter Angabe von absoluten und relativen Häufigkeiten bzw. deren Mittelwert und Standardabweichung. Die Daten wurden mit Microsoft Excel 2021® (Microsoft, USA) erfasst und anschließend mit der Statistiksoftware SPSS® Version 26.0 (IBM, USA) analysiert.

## Ergebnisse

### Studienpopulation

Von den 800 eingeladenen Anästhesisten erhielten wir innerhalb von 2 Monaten nach Studienbeginn 310 ausgefüllte Fragebogen, was einer Antwortrate von 39 % entspricht. Tab. 1 bietet einen Überblick über Charakteristika teilnehmenden Anästhesisten. Vier Fragebogen wurden ausgeschlossen, da die Pflichtfragen nicht beantwortet wurden, sodass schließlich 306 in die endgültige Auswertung eingingen. Die Mehrheit der Teilnehmer war männlich (63 %) mit einem Durchschnittsalter von 41 Jahren (± 10,4). Sie waren hauptsächlich als Oberärzte (34 %), Ärzte in Weiterbildung (34 %), Fachärzte (25 %) oder Chefärzte (8 %) tätig und arbeiteten in Universitätskliniken (37 %) sowie Häusern der Maximalversorgung (27 %), der Schwerpunktversorgung (19 %) oder Grund- und Regelversorgung (15 %). Die Mehrzahl der Anästhesisten (59 %) besaß die Zusatzbezeichnung Notfallmedizin und ist als Notarzt prähospital tätig oder befand sich in der Weiterbildung zum Notarzt (5 %). Zudem hatten 48 % der Befragten die Zusatzbezeichnung Intensivmedizin oder waren derzeit in der entsprechenden Weiterbildung (4 %).

**Tab. 1:** Basischarakteristika der teilnehmenden Anästhesisten

	% (*n*); gesamt *n*
*Geschlecht*	*n* = 300
Männlich	63 % (189)
Weiblich	36 % (109)
Divers	0,3 % (1)
Keine Antwort	2 % (6)
*Alter (Jahre)*	41 Jahre (± 10,4)
Berufserfahrung*	*n* = 306
Arzt in Weiterbildung	34 % (103)
Facharzt	25 % (77)
Oberarzt	34 % (103)
Chefarzt	8 % (23)
*Versorgungsstufe der Klinik*	*n* = 287
Grund- und Regelversorger	15 % (44)
Maximalversorger	27 % (78)
Universitätsklinikum	37 % (107)
Schwerpunktversorger	19 % (55)
Ambulante Tätigkeit	0,7 % (2)
Keine Antwort	7 % (19)
Zusatzweiterbildung*	*n* = 306
Intensivmedizin	48 % (146)
Aktuell in Weiterbildung: Intensivmedizin	4 % (12)
Notfallmedizin	59 % (182)
Aktuell in Weiterbildung: Notfallmedizin	5 % (14)

*Pflichtfragen

### Strukturelle Berücksichtigung der ApOx am Arbeitsplatz

Ein Großteil der Anästhesisten (74 %) gab an, keine Standardarbeitsanweisungen (SOP) zu Evaluation, Identifikation und Dokumentation von Patienten mit Risiken für das Vorliegen einer verminderten Apnoezeit im Rahmen der Narkoseeinleitung in der Allgemeinanästhesie zu haben. In der vorliegenden Untersuchung wurde festgestellt, dass 14 % der Befragten angaben, eine SOP mit strukturierten Handlungsanweisungen zur ApOx im Rahmen der Narkoseeinleitung in ihrer Klinik zu haben. Weitere 2 % gaben an, sowohl eine SOP in der Klinik als auch im Notarztdienst zu haben. Details zeigt Tab. 2.

**Tab. 2:** Strukturelle Berücksichtigung der apnoischen Oxygenierung am Arbeitsplatz

	% (*n*)
*Standardarbeitsanweisungen (SOP) zu Evaluation, Identifikation und Dokumentation von Patienten mit Risiken für das Vorliegen einer verminderten Apnoezeit im Rahmen der Narkoseeinleitung*	*n* = 287
Nein	74 % (212)
Ja	26 % (75)
Keine Antwort	7 % (19)
*Gibt es in Ihrem Krankenhaus eine SOP mit strukturierten Handlungsanweisungen hinsichtlich der apnoischen Oxygenierung im Rahmen der Narkoseeinleitung?*	*n* = 287
Nein	84 % (241)
Ja	14 % (40)
Ja (Notarztdienst)	0 % (0)
Ja (Klinik und Notarztdienst)	2 % (6)
Keine Antwort	7 % (19)

**Tab. 3:** Details zur Anwendung der apnoischen Oxygenierung

	% (*n*)
*Bei welcher Patientengruppe wird die apnoische Oxygenierung standardmäßig durchgeführt?*	*n* = 288
Säuglinge/Kinder	23 % (66)
Erwartet schwieriger Atemweg	42 % (121)
Respiratorische Insuffizienz	54 % (155)
Rapid Sequence Intubation (RSI), Erwachsene	34 % (99)
Rapid Sequence Intubation (RSI), Kinder	23 % (66)
Keine Antwort	6 % (18)
*Sind Sie mit den Indikationen sowie dem Prozedere der apnoischen Oxygenierung im Rahmen der Narkoseeinleitung beim erwachsenen Patienten vertraut?*	*n* = 274
Ja (in Theorie und Praxis)	61 % (166)
Ja (in Theorie)	24 % (66)
Nein	15 % (42)
Keine Antwort	12 % (32)
*Wie häufig haben Sie persönlich die apnoische Oxygenierung bei Erwachsenen durchgeführt?*	*n* = 274
Noch nie	18 % (48)
Sehr selten	26 % (72)
Selten	18 % (49)
Gelegentlich	21 % (57)
Regelmäßig	18 % (48)
Keine Antwort	12 % (32)
*Sofern Sie die apnoische Oxygenierung in Ihrem klinischen Alltag implementiert haben, wie führen Sie diese durch?*	*n* = 276
Nasenbrille	33 % (91)
High-Flow Nasal Cannula (HFNC)	51 % (142)
Rachentubus/Absaugkatheter	10 % (27)
Sonstiges	16 % (44)
Keine Antwort	11 % (30)
*Welche Flussrate wählen Sie für die Sauerstoffzufuhr bei der apnoischen Oxygenierung vor Narkoseeinleitung bei Erwachsenen?*	*n* = 272
6 l/min	5 % (14)
8 l/min	7 % (18)
12 l/min	6 % (16)
15 l/min	21 % (57)
High-Flow Nasal Cannula < 60 l/min, F_I_O_2_ 1,0	14 % (39)
High-Flow Nasal Cannula > 60 l/min, F_I_O_2_ 1,0	35 % (95)
Sonstiges	12 % (33)
Keine Antwort	13 % (34)

**Tab. 4:** Komplikationen bei der Durchführung der apnoischen Oxygenierung

	% (*n*)
*Befürchten Sie bei der Durchführung der apnoischen Oxygenierung im Rahmen der Narkoseeinleitung Komplikationen, die durch diese Methode hervorgerufen werden könnten?*	*n* = 238
Nein	69 % (165)
Ja	9 % (22)
Ich bin mir nicht sicher	21 % (51)
Keine Antwort	29 % (68)
*Welche Art von Komplikationen ist aus Ihrer Sicht bei der Durchführung der apnoischen Oxygenierung relevant?*	*n* = 239
Hypoxie: unzureichende Sauerstoffversorgung trotz dieser Methode	19 % (45)
Hyperkapnie: Anstieg des CO_2_-Gehalts im Blut, da der CO_2_-Abtransport nicht gewährleistet ist	20 % (47)
Aspirationsgefahr	9 % (22)
Barotrauma: Verletzung durch hohen Druck in den Atemwegen	14 % (34)
Schleimhautreizungen: trockene oder gereizte Schleimhäute durch die Sauerstoffzufuhr	24 % (57)
Keine Komplikationen	45 % (107)
Sonstige	6 % (14)
Keine Antwort	28 % (67)

### Details der technischen Anwendung der apnoischen Oxygenierung

Bei der Frage zur Patientengruppe, bei der die ApOx standardmäßig durchgeführt wird, waren Mehrfachnennungen möglich. Die Risikogruppe der Patienten mit einer respiratorischen Insuffizienz wurde am häufigsten genannt (54 %), gefolgt von der Risikogruppe erwarteter schwieriger Atemweg (42 %) und der Rapid Sequence Intubation (RSI) bei Erwachsenen (34 %) sowie RSI bei Kindern und Säuglinge/Kinder allgemein (23 %). Gemäß den Ergebnissen der Studie gaben mehr als die Hälfte der Befragten (61 %) an, sowohl theoretisch als auch praktisch mit den Indikationen und dem Prozedere vertraut zu sein. Es konnte festgestellt werden, dass 15 % der Anästhesisten die Indikationen und das Prozedere der ApOx im Rahmen der Narkoseeinleitung beim Erwachsenen nicht kannten. Eine weitere Gruppe von 24 % der Befragten gab an, nur theoretisches Wissen zu haben. 26 % der Anästhesisten gaben an, die ApOx beim Erwachsenen bisher sehr selten durchgeführt zu haben, 21 % gelegentlich, 18 % selten und 18 % entweder regelmäßig oder noch nie (Tab. 3). Die größte Gruppe an Anästhesisten (51 %) gab an, eine HFNC zu nutzen, 33 % nutzten eine Standardnnasenbrille, 16 % Sonstiges und 10 % einen Rachentubus bzw. Absaugkatheter. Die Flussraten während der ApOx wurden am häufigsten (35 %) als HFNC > 60 l/min, F_I_O_2_ 1,0 gewählt, gefolgt von 15 l/min (21 %), und HFNC < 60l/min, F_I_O_2_ 1,0 (14 %) sowie Sonstigem (12 %).

### Vermutete Komplikationen bei der Anwendung der apnoischen Oxygenierung

Komplikationen bei der Durchführung der ApOx im Rahmen der Narkoseeinleitung, die durch diese Methode hervorgerufen werden, befürchteten 9 % der Anästhesisten, 69 % befürchteten keine Komplikationen, 21 % waren sich nicht sicher (Tab. 4). Bei der Frage, welche Komplikationen den Anästhesisten als relevant erscheinen, waren Mehrfachnennungen möglich. Eine Anzahl von 24 % sahen das Risiko für Schleimhautreizungen, 20 % befürchteten eine Hyperkapnie und 19 % eine Hypoxie trotz Durchführung der ApOx, gefolgt von Barotrauma 14 % und Aspirationsgefahr (9 %).

## Diskussion

Die apnoische Oxygenierung stellt ein Verfahren dar, welches im Rahmen der Narkoseeinleitung zur Verlängerung der sicheren Apnoezeit sowie zur Vermeidung von Hypoxämie zum Einsatz kommt. Trotz Erwähnung in nationalen sowie internationalen Leitlinien ist die praktische Anwendung der ApOx in Deutschland bisher nicht evaluiert [5].

### Studienpopulation und Implikationen

Die Response-Rate von 39 % lag im üblichen Bereich im Vergleich zu ähnlichen Umfragen und sichert eine angemessene Datenbasis [21, 22]. Mit 306 Anästhesisten aus verschiedenen Kompetenzstufen und unterschiedlichen klinischen Versorgungsstufen (Universitätskliniken, Maximalversorger, Schwerpunktversorger, Grund- und Regelversorger) stellt die Stichprobe ein repräsentatives Bild der anästhesiologischen Versorgungsstruktur in Deutschland dar. Der hohe Anteil an Notärzten (59 %) und Intensivmedizinern (48 %) ermöglicht zudem aussagekräftige Schlussfolgerungen für die Notfall- und Intensivmedizin. Gerade bei kritisch kranken Patienten besteht im Rahmen der Atemwegssicherung ein erhöhtes Risiko, den bereits gestörten physiologischen Zustand durch Hypoxämie und Hypotonie während der Einleitung einer Allgemeinanästhesie weiter zu aggravieren [23]. So tritt beispielsweise ein Herzstillstand bei der Notfallintubation außerhalb des OP deutlich häufiger auf [24]. In einer Studie von Sakles et al. wurden die Auswirkungen der ApOx im Rahmen einer RSI auf den First-pass intubation success (FPS) bei 635 Patienten aus einer Notaufnahme untersucht. In dieser Studie wurde der FPS wie folgt definiert: erfolgreiche tracheale Intubation bei einmaligem Einführen des Laryngoskops, ohne dass die Sauerstoffsättigung unter 90 % fällt. In der ApOx-Kohorte konnte ein FPS von 82,1 % registriert werden, im Vergleich zu einer FPS-Rate von 69 % in der Kontrollgruppe (keine ApOx) [25]. In der multivariaten logistischen Regressionsanalyse zeigte sich die Verwendung der ApOx mit einer erhöhten Wahrscheinlichkeit eines FPS („odds ratio“ = 2,2, 95 %-KI = 1,5–3,3). Somit scheint die Anwendung der ApOx auch klinisch relevante „Nebeneffekte“ zu haben. Die genannten Ergebnisse von Sakles et al. deuten darauf hin, dass die Verwendung der ApOx das Potenzial hat, die Sicherheit der RSI zu erhöhen, indem die Anzahl der Intubationsversuche und die Inzidenz von Hypoxämie reduziert werden. Somit lässt sich festhalten, dass die Anwendung der ApOx insbesondere bei Notfallpatienten einen bedeutsamen Beitrag zur Verlängerung der sicheren Apnoezeit sowie der Inzidenz von Desaturationen leisten kann.

### Strukturelle Berücksichtigung der ApOx am Anästhesiearbeitsplatz

SOP dienen dazu, die einheitliche Durchführung definierter Abläufe sicherzustellen. Dadurch wird die Handlungssicherheit für das medizinische Personal, wie beispielsweise im vorliegenden Fall, das an der Narkoseeinleitung beteiligte Anästhesiepersonal erhöht [26]. Die Befragung ergab, dass 74 % der Anästhesisten keine SOP zu Identifizierung und Dokumentation von Patienten mit verkürzter Apnoezeit bei der Narkoseeinleitung verwenden. Dies könnte zum einen daran liegen, dass nicht in allen Krankenhäusern eine SOP zur Sicherung der Atemwege vorhanden ist oder die vorhandenen SOP die Thematik der „Apnoezeit“ nicht adressieren. Aus Sicht der Autoren könnte durch die Einführung einer SOP, welche verschiedene Risikopatientengruppen benennt, eine „situative Awareness“ geschaffen werden, um das Hypoxämierisiko strukturiert einschätzen zu können. Lediglich ein Anteil von 14 % der Anästhesisten hatte in deren Kliniken Zugriff auf eine SOP zur Anwendung der ApOx. Diese Tatsache lässt den Schluss zu, dass die standardisierte Etablierung der ApOx bisher nur auf einem niedrigen Niveau im Klinikalltag in Deutschland verbreitet ist. Unsere Umfrage zeigt auch, dass lediglich 18 % der Anästhesisten angaben, die ApOx regelmäßig durchzuführen. Dies legt nahe, dass die tatsächliche routinemäßige, innerklinische Anwendung der ApOx in Deutschland derzeit noch eine Seltenheit darstellt.

### Einzelheiten zur Durchführung der apnoischen Oxygenierung

Verschiedenen internationale Leitlinien empfehlen die Anwendung der ApOx, wenn eine reduzierte sichere Apnoezeit oder ein hohes Risiko für eine Hypoxämie während der Narkoseeinleitung besteht [4, 5]. Aus diesen Formulierungen geht leider nicht eindeutig hervor, welche Patientengruppen genau gemeint sind. Die dargelegten Umstände bekräftigen die von den Autoren geforderte Einführung von SOP sowie die aktive Deklaration von Risikogruppen, die prädisponiert sind für eine reduzierte Apnoezeit oder Hypoxämie im Rahmen der Narkoseeinleitung. Dadurch könnten Risikogruppen strukturierter erkannt und die ApOx initiiert werden. Die Ergebnisse unserer Studie zeigen, dass ApOx am häufigsten bei Patienten mit respiratorischer Insuffizienz eingesetzt wird. Dies zeigt, dass offensichtlich bei einer bereits bestehenden respiratorischen Insuffizienz die Notwendigkeit einer „zusätzlichen Sicherheitsoption“ durch die Anästhesisten als relevant erachtet wird. Allerdings deutet die aktuelle Datenlage darauf hin, dass die Anwendung von ApOx bei bereits entsättigten Patienten nur von begrenztem Nutzen zu sein scheint [4, 13].

Eine Patientengruppe, für die eine Anwendung der ApOx potenziell von Vorteil sein könnte, sind Patienten mit einem „erwartet schwierigen Atemweg“. Obgleich eine adäquate Vorbereitung erfolgt ist, kann es bei diesen Patienten zu prolongierten Versuchen der Atemwegssicherung kommen. Hier könnte durch die Anwendung der ApOx Zeit „erkauft“ werden, um eine Atemwegsicherung ohne Hypoxämie zu gewährleisten. Dennoch ist es unerlässlich, sämtliche Rahmenbedingungen vor der Einleitung der Anästhesie zu optimieren. Beispielsweise hängt der Erfolg der ApOx maßgeblich von der Qualität der präventiven Präoxygenierung bzw. Denitrogenisierung des Alveolarraums ab. Hohe Partialdrücke anderer Gase (z. B. CO_2_, Stickstoff) beeinträchtigen die für die apnoische Oxygenierung wesentliche Einwärtsdiffusion erheblich [18]. Folglich ist der Effekt der ApOx ist bei Patienten mit eingeschränkter Lungenfunktion oder gestörter alveolärer Diffusion deutlich vermindert [27, 28].

Trotz der Angabe, dass 61 % der Anästhesisten sowohl theoretisch als auch praktisch mit der Anwendung der ApOx vertraut sind, scheint die Anwendung in der klinischen Praxis aktuell noch selten zu sein. Die Ergebnisse der vorliegenden Studie zeigen, dass die Häufigkeit der Anwendung unter den Anästhesisten stark variiert.

Es ist bemerkenswert, dass in 51 % der Fälle, in denen eine ApOx im klinischen Alltag angewendet wird, eine HFNC zum Einsatz kommt, während lediglich in 33 % der Fälle eine Standardnnasenbrille verwendet wird. Insbesondere aus der Kinderanästhesie ist die Verwendung einer HFNC bereits seit Jahren bekannt, stellt jedoch in der Anästhesie von Erwachsenen keinen Standard dar [29]. Im Rahmen dieser Studie wurde nicht explizit geprüft, in welchem Umfeld die Anwendung der HFNC geschieht, jedoch aus Erfahrung der Autoren kann berichtet werden, dass HFNC in Deutschland eher auf der Intensivstation als im OP verfügbar ist. Für bestimmte Risikogruppen kann die Etablierung von HFNC auch im OP von Vorteil sein. Unter Berücksichtigung der Wirtschaftlichkeit und Effizienz erscheint jedoch die Anwendung einer Standardnasenbrille im OP-Bereich eine praktikablere und ubiquitär verfügbare Methode zu sein. Insbesondere in Bereichen der Akut- und Notfallmedizin stehen aktuell keine Geräte zur Anwendung einer HFNC-Therapie zur Verfügung. Unterschiedliche Ansätze und spezifische Anwendervorlieben bei der Durchführung der ApOx können jedoch insbesondere unerfahrene Anwender herausfordern. Die heterogenen Daten legen nahe, dass die Etablierung einer klaren SOP notwendig ist, um bestehende Unklarheiten und Durchführungshindernisse zu überwinden.

### Mit der apnoischen Oxygenierung assoziierte Komplikationen

Die Ergebnisse der Studie legen nahe, dass insgesamt nur 9 % der Anästhesisten Komplikationen durch die Anwendung der ApOx im Rahmen der Allgemeinanästhesie befürchten. Hinsichtlich potenzieller Komplikationen, die durch die hohen Sauerstoffflussraten begründet werden, gaben die Befragten Schleimhautreizungen als mögliche Komplikationen an. Dieser Aspekt lässt sich auch im Umfeld der Autoren immer wieder beobachten. Es wird beispielsweise postuliert, dass die Anwendung von 15 l/min Sauerstoff über die Nase als äußerst unangenehm von den Patienten empfunden wird. Diese Vorbehalte sind allerdings eher persönlicher und anekdotischer Natur und lassen sich zum aktuellen Zeitpunkt wissenschaftlich nicht bestätigen [13, 15]. Ein Kompromiss wäre es, den Sauerstofffluss von 15 l/min über die Nasenbrille erst nach Induktion der Anästhesie freigegeben wird, sofern der „wache“ Patient diesen als unangenehm empfindet [30]. Des Weiteren wurde im Rahmen der Anwendung der ApOx die Sorge vor einer Hyperkapnie berichtet, welche sich aufgrund der fehlenden Ventilation erklären lässt. Dieser Aspekt ist allerdings im Zuge der Anwendung der ApOx für die Narkoseeinleitung von untergeordneter Bedeutung. Die Relevanz dieser Problematik zeigt sich eher dann, wenn die prolongierte ApOx für beispielsweise Operationen/Eingriffe im HNO-Bereich in absoluter Apnoe durchgeführt werden.

In Übereinstimmung mit den Ergebnissen der vorliegenden Untersuchung werden in der Literatur lediglich wenige „Nebeneffekte“ bei der Anwendung der ApOx beschrieben [13, 31]. Dazu zählen beispielsweise mechanische Behinderungen der manuellen Maskenbeatmung oder des Intubationsvorgangs durch die anliegende Nasenbrille [18]. Sollte bei anliegender Nasenbrille keine adäquate Maskendichtigkeit erzielt werden können, kann die Nasenbrille entfernt und erst nach Ende der Beatmung, kurz vor Beginn der Laryngoskopie, durch eine Assistenzperson angelegt werden. Daher sollte die Nasenbrille bereits initial mit dem Anschlussende hinter dem Kopf nach kranial ausgeleitet werden, um ein schnelles Entfernen zu gewährleisten [18]. Insgesamt wird die apnoische Oxygenierung jedoch als sicher und komplikationsarm bewertet und gilt als wertvolles Verfahren zur Vermeidung schwerwiegender Komplikationen im Rahmen des Atemwegsmanagements [4, 12, 18, 30]. Eine intensivere Risikobewertung könnte hier das Sicherheitsgefühl des medizinischen Personals bei der Durchführung der ApOx stärken. Zusätzlich sind weitere Studien nötig, um die Effektivität der ApOx weiter zu bestätigen und dadurch mit höhergradigen Leitlinien Empfehlungen in die klinische Praxis zu integrieren. Beispielsweise wird in den 2024 veröffentlichten europäischen Leitlinien zum Atemwegsmanagement von Neugeborenen und Säuglingen die Anwendung der ApOx bereits favorisiert und abhängig von der Erfahrung des Anwenders empfohlen [32].

### Limitationen

Die vorliegende Studie weist einige relevante Einschränkungen auf: Erstens basieren die Daten ausschließlich auf Fragebogen und spiegeln daher persönliche, subjektive Meinungen wider. Einige Formulierungen von Methoden wurden nicht explizit definiert und können daher von den Anästhesisten unterschiedlich interpretiert werden. Wie bei allen Online-Umfragen garantiert die Anonymität nicht die Richtigkeit der angegebenen Informationen, und eine wiederholte Teilnahme an der Umfrage kann nicht mit absoluter Sicherheit ausgeschlossen werden. In der vorliegenden Untersuchung waren die meisten Teilnehmenden Fachärzte für Anästhesiologie, somit stellen Ärzte in Weiterbildung eine Minderheit dar. Hinsichtlich der gegenwärtigen Anwendungspraxis der ApOx in Deutschland liegen keine weiteren Daten vor. Deshalb konnten die Ergebnisse nicht mit den Daten anderer Untersuchungen zu diesem Thema verglichen werden. Die angewandte Umfragemethode erlaubt keine präzise Abschätzung der Anzahl der Personen, die eine Einladung tatsächlich persönlich erhalten haben. Die Zahl von 800 Einladungen ist daher eine errechnete Zahl, die auf der Anzahl der Mitarbeiter in den verschiedenen Kliniken sowie auf der Anzahl der Personen im DGAI-Verteiler des Wissenschaftlichen Arbeitskreises Atemwegsmanagement basiert.

## Schlussfolgerung

Die vorliegende Studie liefert erstmalig einen Einblick in den aktuellen Stand der praktischen Anwendung der ApOx in deutschen anästhesiologischen Kliniken. Mit einer Stichprobe verschiedener Erfahrungsstufen und aus unterschiedlichen klinischen Versorgungsstufen konnte ein umfassendes Bild der gegenwärtigen Praxis gewonnen werden. Die Mehrheit der befragten Anästhesisten verfügt über keine strukturierten SOP zu Identifizierung und Behandlung von Patienten mit potenziell verkürzter sicherer Apnoezeit an ihrem Arbeitsplatz. Obwohl die ApOx bei Patienten mit respiratorischer Insuffizienz und schwierigen Atemwegsbedingungen eingesetzt wird, zeichnen sich in dieser Umfrage sichtbare Unterschiede in den Kenntnissen und der Art und Weise der praktischen Anwendung ab. Hinsichtlich der applizierten Sauerstoffflussrate lässt sich keine Standardisierung feststellen. Dies zeigt, dass weitere Maßnahmen notwendig sind, um die praktische Anwendung und das theoretische Wissen besser aufeinander abzustimmen. Hinsichtlich der Komplikationen befürchtete nur eine Minderheit der Anästhesisten spezifische Risiken wie z. B. Schleimhautreizungen, Hyperkapnie oder Hypoxie bei Anwendung der ApOx. Diese geringe wahrgenommene Komplikationsrate sollte eine weitere Verbreitung der ApOx befürworten. Zukünftige Forschungen sollten sich darauf konzentrieren, die ApOx-Strategien weiter zu evaluieren und ihre Anwendung und Effektivität in vulnerablen Patientengruppen zu erfassen.

## Fazit für die Praxis

- Obgleich in der Literatur eine signifikant geringere Hypoxämierate sowie eine gesteigerte Wahrscheinlichkeit für einen First-pass intubation success bei Anwendung der ApOx beschrieben werden, findet die Methode in Deutschland derzeit keine routinemäßige Anwendung.
- Die flächendeckende Implementierung der ApOx, insbesondere im Kontext der Notfallnarkose, eröffnet die Möglichkeit einer Optimierung der Patientensicherheit für eine vulnerable Patientengruppe.
- Hinsichtlich der angewandten Sauerstoffflussraten zur ApOx existiert kein standardisiertes Vorgehen.
- Die ApOx ist nicht als Gamechanger zu verstehen, sondern als eine Methode, um die Apnoezeit bei hypoxämiegefährdeten Patienten ohne Desaturation zu verlängern.

## Supplementary Information

Fragenkatalog zum Online Survey Apnoische Oxygenierung, Version 1.0, Stand 01.06.2024

## Data Availability

Die im Rahmen der Studie erfassten Daten können in begründeten Fällen vom korrespondierenden Autor erhalten werden.
