# Supplementary material for: Utilization of apneic oxygenation in anesthesiology: a nationwide survey in Germany
Source: Anaesthesiologie. 2025 Apr 28;74(5):283–93. [Article in German] doi: 10.1007/s00101-025-01529-2 (PMC12081577; doi:10.1007/s00101-025-01529-2)
Supplement: Supplementary file 1 — Fragenkatalog zum Online Survey Apnoische Oxygenierung, Version 1.0, Stand 01.06.2024 [file 101_2025_1529_MOESM1_ESM.pdf]

Verantwortlicher Studienleiter:

Dr. med. D.D. Uzun , Klinik für Anästhesiologie Universitätsklinikum Heidelberg

---

Anschreiben

Wir bitten um Ihre Teilnahme an der Umfrage zur Apnoeischen Oxygenierung in der Anästhesiologie.

Sehr geehrte Kolleginnen und Kollegen,

wir möchten Sie herzlich einladen, an unserer Umfrage „Apnoeische Oxygenierung in der Anästhesiologie“ teilzunehmen, die sich mit den folgenden Kernfragen befasst:

Welchen Stellenwert hat die apnoeische Oxygenierung bei Narkoseeinleitungen im klinischen Alltag an deutschen Kliniken, wie regelmäßig wird sie angewendet, wie genau wird sie durchgeführt und bei welchen Patientengruppen wird eine verlängerte sicher Apnoezeit als wichtig erachtet.

Unser Ziel ist es, einen Überblick über die Praxis der apnoeischen Oxygenierung in Deutschland, ihre Indikationen und ihr klinisches Management zu erhalten.

Beginn Survey

Sehr geehrte Kolleginnen und Kollegen,

herzlich willkommen zu unserer online Umfrage „Apnoeische Oxygenierung in der Anästhesiologie“.

Wir freuen uns sehr über Ihr Interesse. Mit Ihrer Teilnahme leisten Sie einen wertvollen Beitrag, den Standard bei der Narkoseeinleitung von Risikopatienten zu skizzieren und Ansätze zur Verbesserung und Optimierung der medizinischen Versorgung zu identifizieren.

Die Beantwortung des Fragebogens wird etwa 5 min Ihrer Zeit in Anspruch nehmen. Die online Umfrage ist vollständig anonym und freiwillig. Durch die Ethikkommission der Medizinischen Fakultät der Universität Heidelberg wurden keinerlei Bedenken zur vorliegenden Umfrage geäußert.

Vielen dank im Voraus für Ihre Unterstützung und Ihre Zeit.

Für weitere Informationen oder Rückfragen stehen die Kollegen der Klinik für Anästhesiologie am Universitätsklinikum Heidelberg jederzeit zur Verfügung.

Ansprechpartner zur Umfrage:

Dr. med. D. D. Uzun

Deniz.Uzun@med.uni-heidelberg.de

Arzt der Klinik für Anästhesiologie (Ärztlicher Direktor Prof. Dr. med. M. A. Weigand)

Universitätsklinikum Heidelberg

69120 Heidelberg

und

PD Dr. med. F. Schmitt, MHBA, DESAIC

Oberarzt der Klinik für Anästhesiologie (Ärztlicher Direktor Prof. Dr. med. M. A. Weigand)

Universitätsklinikum Heidelberg

69120 Heidelberg

* Zur besseren Lesbarkeit wird in dieser Umfrage das generische Maskulinum verwendet. Die in dieser Arbeit verwendeten Personenbezeichnungen beziehen sich – sofern nicht anders kenntlich gemacht – auf alle Geschlechter.

1. Bitte geben Sie Ihr Alter an:

2. Was trifft auf Sie zu?
   a. Weiblich
   b. Männlich
   c. Divers

3. Welchen Ausbildungsstand haben Sie?
   a. Arzt in Weiterbildung
      a. 1. Jahr
      b. 2. Jahr
      c. 3.Jahr
      d. 4. Jahr
      e. 5. Jahr
      f. >5. Jahr
   b. Facharzt
      a. <5 Jahre als Facharzt tätig
      b. >5 Jahre als Facharzt tätig
   c. Oberarzt
   d. Chefarzt

4. In welche Versorgungstufe lässt sich Ihr Hauptarbeitsplatz einteilen?
   a. Grund- und Regelversorger
   b. Maximalversorger
   c. Universitätsklinikum
   d. Schwerpunktversorger
   d. Ambulante Tätigkeit

5. Besitzen Sie die Zusatzbezeichnung Notfallmedizin?
      a. Ja
      b. Nein
      c. Zur Zeit in Weiterbildung

6. Besitzen Sie die Zusatzbezeichnung Intensivmedizin?
      d. Ja
      e. Nein
      f. Zur Zeit in Weiterbildung

7. Existieren in Ihrem Krankenhaus strukturierte Pfade (SOP) zur Evaluation, Identifikation und Dokumentation von Patienten mit Risiken für das Vorliegen einer verminderten Apnoezeit im Rahmen der Narkoseeinleitung?

   a. Nein
   b. Ja

8. Gibt es in Ihrem Krankenhaus einen Versorgungstandard (SOP) mit strukturierten Handlungsanweisungen hinsichtlich der apnoischen Oxygenierung im Rahmen der Narkoseeinleitung
   a. Nein
   b. Ja (Klinik)
   c. Ja (Notarztdienst)
   d. Ja (Klinik + Notarztdienst)

9. Bei welcher Patientengruppe wird die apnoeische Oxygenierung standardmäßig durchgeführt?
   a. Säuglinge/Kinder
   b. Risikogruppen:
      i. erwartet schwieriger Atemweg,
      ii. Respiratorische Insuffizienz,
      iii. Rapid Sequence Induction (RSI) Erwachsene
      iv. Rapid Sequence Induction (RSI) Kinder

10. Sind Sie mit den Indikationen sowie dem Procedere der apnoeischen Oxygenierung im Rahmen der Narkoseeinleitung beim erwachsenen Patienten vertraut?
    a. Ja (in Theorie und Praxis)
    b. Ja (in Theorie)
    c. Nein

11. Wie häufig haben Sie persönlich eine apnoeische Oxygenierung bei Erwachsenen durchgeführt?
    a. Noch nie
    b. Sehr selten
    c. Selten
    d. Gelegentlich
    e. Regelmäßig

12. Sofern Sie die apnoeische Oxygenierung in Ihrem klinischen Alltag implementiert haben, wie führen Sie diese durch?
    a. Nasenbrille
    b. High-Flow-Nasal-Cannula (HFNC)
    c. Rachentubus/Absaugkatheter
    d. Sonstiges

13. Welche Flussrate wählen Sie für die Sauerstoffzufuhr bei der apnoeischen Oxygenierung vor der Narkoseeinleitung bei erwachsenen Patienten?
    a. 6L/min
    b. 8L/min
    c. 12L/min
    d. 15L/min
    e. High-Flow-Nasal-Cannula <60l/min, FiO_2 1,0
    f. High-Flow-Nasal-Cannula >60l/min, FiO_2 1,0
    g. Sonstiges

14. Befürchten Sie bei der Durchführung der apnoeischen Oxygenierung im Rahmen einer Narkoseeinleitung Komplikationen, die durch diese Methode hervorgerufen werden könnten?
    a. Ja
    b. Nein
    c. Ich bin mir nicht sicher

15. Welche Art von Komplikationen sind aus Ihrer Sicht bei der Durchführung der apnoeischen Oxygenierung relevant?
    a. Hypoxie: Unzureichende Sauerstoffversorgung trotz der Methode
    b. Hyperkapnie: Anstieg des Kohlendioxidgehalts im Blut, da der CO_2-Abtransport nicht gewährleistet ist
    c. Aspirationsgefahr
    d. Barotrauma: Verletzungen durch hohen Druck in den Atemwegen
    e. Schleimhautreizungen: Trockene oder gereizte Schleimhäute durch die Sauerstoffzufuhr
    f. Sonstiges

16. Freitext:
